# Surface Carrier Testing of Hospital Antiseptics Against *Candida parapsilosis* from Healthcare Workers’ Hands

**DOI:** 10.3390/pathogens15040410

**Published:** 2026-04-10

**Authors:** Jenyffie Araújo Belizário, Maria Eduarda Brites Jardine, Gabrielle Lameado Pereira, Murilo Molina Stefani, Ralciane de Paula Menezes, Denise von Dolinger de Brito Röder, Reginaldo dos Santos Pedroso, Sérgio Ricardo Ambrósio, Gil Benard, Regina Helena Pires

**Affiliations:** 1 Laboratory of Microbiology, University of Franca, Franca 14404-600, SP, Brazil; jeeabelizario@gmail.com (J.A.B.); mariabritesedu@gmail.com (M.E.B.J.); gabriellelameado@hotmail.com (G.L.P.); murilomolina2@gmail.com (M.M.S.); sergio.ambrosio@unifran.edu.br (S.R.A.); 2 Institute of Biomedical Sciences, Federal University of Uberlândia, Uberlândia 38408-100, MG, Brazil; ralciane@ufu.br (R.d.P.M.); denise.roder@ufu.br (D.v.D.d.B.R.); 3 Technical School of Health, Federal University of Uberlândia, Uberlândia 38408-100, MG, Brazil; rpedroso@ufu.br; 4 Laboratory of Medical Mycology (LIM-53), Institute of Tropical Medicine, Hospital das Clínicas, Department of Dermatology, Faculty of Medicine, University of São Paulo, São Paulo 05508-220, SP, Brazil

**Keywords:** antiseptics, hand disinfection, chlorhexidine, antifungal agent

## Abstract

*Candida parapsilosis* is a major cause of healthcare-associated infections due to its persistence on abiotic surfaces and efficient transmission via healthcare workers’ hands. This study evaluated the antifungal efficacy and safety of clinically relevant antiseptics against 60 *C. parapsilosis* clinical isolates using a surface carrier test designed to simulate contamination and disinfection events on hospital surfaces. Antifungal activity was assessed by logarithmic reduction (log_10_) assays on surface carriers and by minimum inhibitory concentration (MIC) testing. Potential synergistic interactions between antiseptics and selected phytochemicals were investigated using checkerboard assays, and toxicity was evaluated in vivo using *Caenorhabditis elegans*. Surface carrier assays showed that 70% ethanol and 0.5% alcoholic chlorhexidine (CHG) achieved the highest fungicidal activity, with reductions of up to 5 log_10_ after 1 min exposure at 25 °C. Polyhexamethylene guanidine hydrochloride (PHMGH) displayed consistently low MIC values (0.4–0.9 ppm) and intermediate surface activity. CHG combined with eugenol or menthol produced strong synergistic interactions, reducing CHG MICs from up to 6250 ppm to as low as 20 ppm (>300-fold). Toxicity assays revealed a narrow safety margin for CHG, whereas PHMGH showed a more gradual concentration-dependent toxicity profile. These findings highlight clinically relevant differences in antiseptic performance and identify combination strategies that may reduce CHG exposure while maintaining antifungal efficacy.

## 1. Introduction

*Candida parapsilosis* is a human commensal yeast, frequently acquired from exogenous sources, and exhibits a remarkable capacity to form biofilms on intravascular devices such as catheters, as well as to persist for extended periods on inanimate surfaces, including floors, furniture, and hospital equipment [1,2,3]. Since 2005, *C. parapsilosis* has been reclassified as a complex comprising three phylogenetically related species, *C. parapsilosis* sensu stricto, *C. metapsilosis*, and *C. orthopsilosis*, which are morphologically indistinguishable and require molecular or proteomic approaches for accurate identification [3,4].

In Brazil, *C. parapsilosis* has been identified as a causative agent in recurrent hospital outbreaks, frequently associated with cross-transmission through hand contact between healthcare workers and patients [1,3,5,6]. Epidemiological studies indicate that *C. parapsilosis* is among the leading causes of candidemia in Latin America, in some centers accounting for approximately 20–30% of *Candida* bloodstream infections, particularly in neonatal and intensive care units [2,3]. This pattern of dissemination is aggravated by persistent deficiencies in cleaning and disinfection procedures, as well as limited adherence to effective biosafety protocols. Collectively, these factors contribute to the maintenance of an active epidemiological chain, increasing the risk of cross-contamination involving the hospital environment, healthcare workers, and patients, thereby compromising the quality of care and patient safety [7,8].

In this context, the use of effective biocides constitutes a key strategy for controlling the dissemination of infectious agents in clinical settings. These compounds may act as sanitizers, reducing the microbial load, or as high-level disinfectants, inactivating a broad range of microorganisms through mechanisms that vary according to their chemical composition [9]. When applied to living tissues, they are classified as antiseptics; on inanimate surfaces, they are considered disinfectants [10]. However, antiseptic efficacy is strongly influenced by environmental conditions such as surface adhesion, drying, organic matter, and short contact times, which are not reproduced in conventional planktonic susceptibility assays. As a result, antiseptic performance observed under laboratory conditions may not accurately reflect efficacy during real contamination and disinfection events on hospital surfaces. Among the most widely used active agents in hospitals are quaternary ammonium compounds, benzalkonium chloride, aldehydes, hydrogen peroxide, biguanides, isopropyl alcohol, peracetic acid, and iodophors [11]. More recently, polyhexamethylene guanidine hydrochloride (PHMGH), a member of the polymeric guanidine class, has attracted attention due to its broad antimicrobial activity, including efficacy against biofilm-forming fungi [12,13,14].

However, increasing cross-resistance among hospital-associated microorganisms, including fungal pathogens, has challenged the effectiveness of conventional antimicrobials and antiseptics, highlighting the need for updated protocols and safer, more effective infection control strategies [15]. Traditional hand antisepsis practices, such as washing with soap followed by iodinated or absolute alcohol, although still used in some institutions, may cause skin irritation and lesions, leading to suboptimal adherence and compromised microbial load reduction [16].

In this context, CHG remains one of the most widely used antiseptics in healthcare, applied at various concentrations for hand antisepsis, oral hygiene, and patient bathing [17,18]. Its broad-spectrum activity against Gram-positive and Gram-negative bacteria and yeasts is primarily mediated by disruption of microbial cell membrane integrity, resulting in cell death [18]. PHMGH has emerged as a promising antimicrobial polymer, demonstrating effective fungicidal and bactericidal activity even in the presence of organic matter and biofilms, conditions under which conventional antiseptics often show reduced performance [19,20,21]. In parallel, increasing interest has focused on formulations combining natural phytochemicals such as thymol, eucalyptol, eugenol, and menthol, which exhibit antimicrobial activity and may enhance the efficacy of traditional antiseptics through synergistic interactions [22,23].

Despite the widespread use of antiseptics in healthcare settings, evidence regarding their antifungal activity against *C. parapsilosis* under experimental conditions that simulate contamination and disinfection events on hospital surfaces remains limited. Most available studies evaluate antiseptic efficacy using conventional in vitro susceptibility assays that do not reproduce the conditions under which contamination occurs on abiotic surfaces. Consequently, the effectiveness of antiseptics under realistic surface contamination conditions remains insufficiently understood. Surface carrier–based methods provide a more realistic approach to simulate contamination and disinfection events on hospital surfaces, but such models remain underexplored for *C. parapsilosis*.

Therefore, the present study aimed to evaluate the antifungal activity of commercial antiseptics or their active principles, alone and in combination with phytochemicals, against clinical isolates of *C. parapsilosis* obtained from the hands of healthcare workers using a surface carrier–based exposure model. We hypothesized that assessing antiseptic activity under these conditions, using epidemiologically relevant isolates, would provide more realistic insights into antiseptic performance and reveal potential improvements when combined with phytochemicals. By addressing this methodological gap, this study seeks to contribute to the development of more effective infection control strategies in healthcare environments.

## 2. Materials and Methods

### 2.1. The Antiseptics

The antiseptics evaluated in this study comprised both commercial formulations and pure active ingredients, in accordance with national and international guidelines for hospital antisepsis [24,25]. Pure compounds were used when commercial formulations contained multiple excipients or additional components that could potentially interfere with the experimental analysis or confound the interpretation of results. The tested products included ethanol 70% (EtOH), commercially available as Rialcool 70^®^, a product manufactured by Rioquímica S.A. (São José do Rio Preto, SP, Brazil), which is widely used as a standard antiseptic for hand and skin disinfection. Chlorhexidine digluconate (CHG) was evaluated in three formulations: Riohex Gard 0.12%^®^ (Rioquímica), containing 0.12% CHG, typically used as an oral rinse for mechanically ventilated patients; Riohex 0.5%^®^ alcoholic solution (Rioquímica), containing 0.5% CHG in ethanol. This alcohol-based formulation was included because alcohol is known to provide rapid antimicrobial activity through protein denaturation and membrane damage, while CHG provides residual activity, and the combination is widely used in clinical practice for skin antisepsis. A 1% aqueous solution of CHG (Needs, Raia Drogasil S/A, São Paulo, SP, Brazil) was also tested as a topical antiseptic to allow comparison between alcoholic and aqueous CHG formulations. Hydrogen peroxide 3% (H_2_O_2_) was assessed using a 10-volume pharmaceutical formulation (Rioquímica), and isopropyl alcohol 70% (IPA) was obtained from Cinord Indústria Farmacêutica Ltd.a. (Serrana, SP, Brazil), both used as general-purpose antiseptics. Chloroxylenol (PCMX) was tested at a concentration of 1% (Sigma-Aldrich Co., São Paulo, SP, Brazil), representing a phenolic antiseptic commonly used in hygienic handwashing. Polyhexamethylene guanidine hydrochloride (PHMGH) was evaluated as a 0.4% solution (Akwaton^®^, Nordic Chem, Brampton, ON, Canada), selected based on concentration ranges previously reported in the literature to demonstrate antifungal activity [26,27,28]. Povidone–iodine (PVP-I), in a solution of 10% povidone–iodine and 1% free iodine, was evaluated through the Riodeine^®^ aqueous solution (Rioquímica), traditionally used for skin antisepsis in wound care. Finally, triclosan (TCS), a synthetic broad-spectrum antimicrobial agent, was tested at a concentration of 0.5% (Sigma-Aldrich, code product 72779), commonly used in antiseptic soaps and handwashing formulations due to its anti-biofilm properties. All antiseptics were tested in their undiluted form, in alignment with real-world application practices, and applied according to the manufacturer’s instructions or based on relevant scientific recommendations when applicable.

### 2.2. Yeasts

A total of 60 clinical isolates of *C. parapsilosis* sensu stricto, obtained from the hands of healthcare workers in oncological and neonatal intensive care units in Brazil and cryopreserved, were kindly provided by the Carlos da Silva Lacaz Culture Collection and by Prof. Denise Von Dolinger de Brito Röder, Institute of Biomedical Sciences, Federal University of Uberlândia, MG, Brazil, respectively. All isolates had been previously identified by random amplified polymorphic DNA (RAPD; SADH gene, Ban I enzyme) and by matrix-assisted laser desorption/ionization (MALDI) followed by detection using a time-of-flight (TOF) analyzer, as previously described [1,6,29]. All isolates were confirmed to be biofilm formers and the antifungal susceptibility profiles (azoles, echinocandins, and amphotericin B) of these isolates have been published previously [1,29]. The reference strains *Candida parapsilosis* ATCC 90018 and *Candida albicans* ATCC 10231 [30] were used as quality controls.

### 2.3. Determination of the Fungicidal Activity of Antiseptics Using the Disk-Based Quantitative Carrier Test

The quantitative carrier test based on the disk method [31] was employed, using sterile circular glass coverslips (13 mm diameter) as carriers, placed in Petri dishes. Glass carriers were used as non-porous surfaces because glass is an inert material commonly used in standardized carrier tests for disinfectant evaluation, including European methodologies, allowing reproducible contamination, drying, and microbial recovery. A 10 μL aliquot of *C. parapsilosis* inoculum (0.5 to 1.5 × 10^6^ CFU/mL), prepared in Yeast Peptone Dextrose (YPD) broth supplemented with 3% fetal bovine serum to simulate the presence of organic matter [32], was deposited onto each coverslip and left to dry under laminar airflow for 2 h [33]. Once dried, the inoculated surfaces were exposed to 25 μL of the test antiseptic for 1 min at room temperature [33]. The coverslips were then transferred into sterile Falcon tubes containing 9.75 mL of neutralizing solution composed of 10 g Tween 80 (Sigma-Aldrich, St. Louis, MI, USA), 1 g lecithin (Sigma-Aldrich), 0.5 g L-histidine (Sigma-Aldrich), 2.5 g sodium thiosulfate (Na_2_S_2_O_3_; Avantor, Gliwice, Poland), 3.5 g sodium pyruvate (C_3_H_3_NaO_3_; Avantor), and 1000 mL sterile water [34]. The tubes were vortexed, and 100 μL of each suspension was plated onto YPD agar using a Drigalski loop. After incubation at 36 ± 1 °C for 48 to 72 h, colony-forming units (CFU) were enumerated, and the logarithmic reduction factor (RF) was calculated using the formula:

RF = log_10_ (CFU_control/CFU_treated)

In this equation, CFU_control refers to the mean number of microorganisms recovered from non-exposed biofilms (negative control), while CFU_treated represents the mean count following exposure to the tested antiseptic [32]. An antiseptic was considered effective when it achieved a reduction of ≥4 log_10_ CFU/mL relative to the initial inoculum, in accordance with current international standards for bactericidal and yeasticidal efficacy in the presence of organic matter, as defined in EN 17387:2021 [31]. Results were reported with two decimal places, and biological replicates were considered valid when variability among them did not exceed 1 log_10_ [30]. All tests were performed in triplicate.

### 2.4. Determination of MIC and the Combinatory Effect Between Antiseptics and Phytochemicals

Twelve clinical isolates were randomly selected for these assays. Checkerboard experiments were performed exclusively with CHG 0.5% in alcoholic solution and PHMGH. CHG and PHMGH were selected as representative antiseptics for further analysis, reflecting their established clinical use and emerging antimicrobial relevance, respectively [26,28,35,36,37]. In addition, four phytochemicals with recognized antimicrobial properties, including eugenol, eucalyptol, menthol, and thymol, were tested. Although these compounds are commonly used in mouthwash formulations [22], they have not yet been incorporated into topical antiseptic products for skin antisepsis in Brazil. The antifungal activity of CHG or PHMGH in combination with phytochemicals was evaluated using the broth microdilution checkerboard method according to EUCAST guidelines [38]. Fungal suspensions were adjusted to 2.5 × 10^5^ cells/mL. MICs of CHG and PHMGH alone were determined under the same conditions. After incubation, growth was assessed using resazurin as a colorimetric viability indicator, and MIC was defined as the lowest concentration preventing the color change from blue to pink. Drug interactions were evaluated by calculating the fractional inhibitory concentration index (FICI), defined as ΣFIC = FIC_A + FIC_B, where each FIC corresponds to the MIC of the drug in combination divided by the MIC of the drug alone [39,40]. Results were interpreted as follows: FICI ≤ 0.5 indicated synergy; values > 0.5 and ≤4.0 indicated indifference; and FICI > 4.0 indicated antagonism. All phytochemicals were purchased from Sigma-Aldrich (São Paulo, SP, Brazil) with certified purity levels: thymol (98.5%), eucalyptol (99%), menthol (99%), and eugenol (98%).

### 2.5. Toxicity Assessment in Caenorhabditis elegans

L4 larvae of the *C. elegans* AU37 strain [glp-4(bn2) I; sek-1(km4) X] were cultured on plates containing Nematode Growth Medium (NGM) seeded with *Escherichia coli* OP50 at 16 °C. Synchronization was achieved by alkaline hypochlorite treatment to obtain eggs, which hatched overnight in M9 buffer to produce synchronized L1 larvae. After 48 h of growth on NGM, L4-stage larvae were washed with M9 buffer and transferred to 96-well microplates (approximately 20 worms per well) containing liquid medium supplemented with cholesterol (10 µg/mL), kanamycin (90 µg/mL) and ampicillin (200 µg/mL) [41]. Only individual compounds were tested, and the concentrations were selected based on MIC values determined for each compound alone or on MIC values obtained in combination from the checkerboard assay (FICI-derived MICs). Microplates were incubated at 25 °C, and larval survival was assessed after 2, 4, 6, 8, and 24 h of exposure by direct observation of the response to gentle mechanical stimulus. Nematodes were considered dead when they exhibited a rigid, rod-like morphology and a complete absence of motility [42]. Amphotericin B (1.0 ppm) and DMSO (1%) were used as controls.

### 2.6. Statistical Methods

Data distribution was examined for homogeneity of variances using Levene’s test, which indicated heteroscedasticity (W = 15.54; *p* < 0.001). Consequently, group comparisons were performed using Welch’s analysis of variance (Welch’s ANOVA), which does not assume equal variances, followed by the Games–Howell post hoc test, a procedure robust to unequal variances and sample sizes. Effect size was estimated using eta squared (η^2^). Statistical significance was set at *p* < 0.05. Results are presented as mean log_10_ reduction with 95% confidence intervals.

## 3. Results

### 3.1. Logarithmic (log_10_) Reduction in C. parapsilosis Cells After Exposure to Different Antiseptics Using a Disk-Based Carrier Model

**Figure 1:**
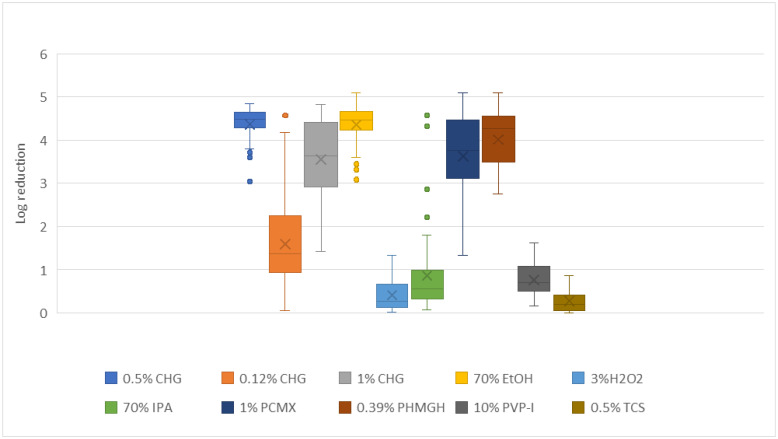
Logarithmic (log_10_) reduction in *C. parapsilosis* cells after exposure to different antiseptics using a disk-based carrier model. Boxplots show the median, interquartile range, and variability of reductions achieved with each compound: 70% ethanol (EtOH), 0.5% chlorhexidine in alcohol (CHG), 0.4% polyhexamethylene guanidine hydrochloride (PHMGH), 1% aqueous chlorhexidine (CHG), 0.12% chlorhexidine (CHG), 0.5% triclosan (TCS), 1% para-chloro-meta-xylenol (PCMX), 70% isopropyl alcohol (IPA), 10% povidone-iodine (PVP-I), 3% hydrogen peroxide (H_2_O_2_)._2_). All experiments were performed in triplicate, and results are presented as mean log_10_ reductions with 95% confidence intervals.

Exposure of isolates to the different antiseptics using a surface carrier–based exposure model, designed to mimic abiotic surface contact, resulted in significant variation in log reduction (Figure 1). EtOH, CHG 0.5%, PCMX, and PHMGH exhibited the highest reductions, with medians close to 4 log_10_ and maximum values reaching up to 5 log_10_, indicating strong effectiveness against *C. parapsilosis* cells. CHG 1% showed intermediate performance, with reductions predominantly ranging between 3 and 4 log_10_, though with greater variability. In contrast, CHG 0.12% displayed more limited efficacy, with reductions mostly around 1 to 2.5 log_10_. Other compounds, including IPA, PVP-I, H_2_O_2_, and TCS, resulted in comparatively low reductions, generally ≤1.5 log_10_, reflecting restricted activity against the organism. Log_10_ reduction values are presented as mean reductions with 95% confidence intervals, representing the overall performance across isolates rather than single maximal values.

**Figure 2:**
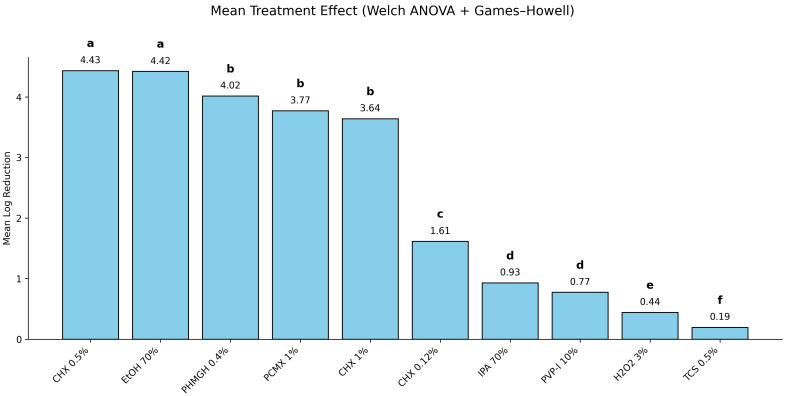
Mean log_10_ reduction in *C. parapsilosis* cells following treatment with different antiseptics. Bars represent mean ± 95% confidence interval. Different letters indicate statistically significant differences between groups according to Welch’s ANOVA followed by Games–Howell post hoc test (*p* < 0.05).

To determine whether these observed differences were statistically robust across treatments, a comparative analysis was performed. The statistical analysis revealed a highly significant effect of treatment on mean log reduction (F(8, 235.22) = 1565.49; *p* < 0.001), with a large effect size (η^2^ = 0.85) (Figure 2). Multiple comparisons (Figure 2) showed that CHG 0.5% and EtOH formed the most effective group (letter “a”), with no significant difference between them. PHMGH, PCMX, and CHX1 (letter “b”) exhibited intermediate efficacy, significantly lower than group “a”.

CHG 0.12% (letter “c”) showed a moderate effect, while IPA and PVPI (letter “d”) and H_2_O_2_ (letter “e”) were among the least effective. TCS (letter “f”) was the least effective treatment overall. According to internationally accepted efficacy thresholds for antiseptics applied to surfaces, these findings indicate that CHG 0.5% and EtOH are the most suitable options for achieving maximal microbial reduction, whereas TCS shows limited performance under surface-associated exposure conditions.

### 3.2. Antifungal Activity and Interaction Profiles of CHG and PHMGH Combined with Phytochemicals

Given these differences in killing efficacy, additional analyses were conducted to further characterize the inhibitory activity of selected antiseptics. The MIC and interaction data for CHG and PHMGH are presented in Table 1 and Table 2, respectively. CHG showed substantially higher and more variable MICs, spanning from 150 to 6250 ppm among the tested isolates (Table 1). In contrast, PHMGH (Table 2) consistently exhibited the lowest MIC values against *C. parapsilosis* clinical isolates, ranging from 0.4 to 0.9 ppm. MICs of 1.9 ppm and 0.9 ppm were observed for *C. albicans* ATCC 10231 and *C. parapsilosis* ATCC 90018, respectively.

Combination assays were subsequently performed to evaluate whether phytochemicals could enhance the antifungal activity of CHG or PHMGH. Checkerboard testing revealed that the association of CHG with eugenol or menthol resulted predominantly in synergistic interactions (FICI ≤ 0.5) across the majority of the 12 *C. parapsilosis* clinical isolates, as well as the reference strains (Table 1). Importantly, these synergistic profiles were accompanied by pronounced reductions in CHG MICs, with fold reductions (FR) ranging from 8- to over 300-fold compared with CHG tested alone, underscoring a substantial gain in antifungal potency not fully reflected by FICI values alone. By contrast, combinations of CHG with eucalyptol or thymol yielded more heterogeneous outcomes. While synergism was observed for several isolates, indifferent interactions (FICI > 0.5–4.0) were also frequent, particularly for the reference strains, and no consistent enhancement of CHG activity was evident for all isolate–compound pairs (Table 1).

For PHMGH, interactions with phytochemicals were predominantly classified as indifferent (FICI > 0.5–4.0) across the majority of *C. parapsilosis* clinical isolates and reference strains (Table 2). In most combinations, PHMGH MIC values remained unchanged, resulting in fold reductions close to 1, indicating limited enhancement of antifungal activity. Notable exceptions were observed for the combination with thymol, which displayed clear synergistic interactions in isolate 2A010 (FICI = 0.06) and in *C. parapsilosis* ATCC 90019 (FICI = 0.03). Additionally, a synergistic interaction was detected for the PHMGH–menthol combination in isolate 3D033 (FICI = 0.5).

### 3.3. Survival and Concentration-Dependent Toxicity of Tested Compounds in Caenorhabditis elegans

**Figure 3:**
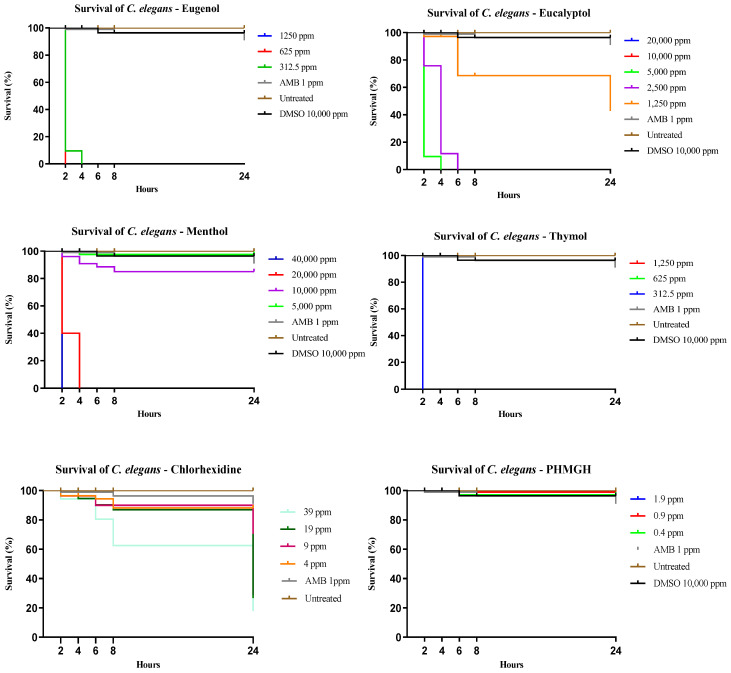
Survival of *Caenohabditis elegans* following exposure to different antiseptics and active compounds at concentrations based on the MIC values determined in this study. All tested concentrations are shown for each compound, except for chlorhexidine, for which concentrations above 39 ppm induced acute toxicity with marked mortality within the first 2 h of exposure and were therefore excluded from graphical representation. Survival was monitored over time, revealing compound- and concentration-dependent effects. AMB, amphotericin B; DMSO, dimethyl sulfoxide; PHMGH, polyhexamethylene guanidine hydrochloride.

Exposure of *C. elegans* to the tested substances revealed distinct, concentration-dependent survival profiles (Figure 3). For CHG, concentrations above 39 ppm induced acute toxicity, with pronounced mortality observed within the first 2 h of exposure. Due to this rapid toxic effect, these higher concentrations were excluded from graphical representation, and only sub-MIC and MIC-related concentrations are shown. In contrast, exposure to PHMGH across the full range of tested concentrations did not result in acute early toxicity. Survival decreased in a concentration-dependent manner but remained comparatively higher at MIC and sub-MIC levels, indicating a more favorable in vivo tolerance relative to CHG under the conditions tested.

For the remaining compounds (Figure 3), *C. elegans* survival was evaluated across the full range of tested concentrations, all of which are presented in the corresponding graphs. For these agents, survival remained high at lower concentrations and decreased progressively with increasing dose, indicating variable but generally more gradual toxic effects when compared with CHG.

## 4. Discussion

The marked differences in log_10_ reduction observed among the tested antiseptics are consistent with recent evidence on antifungal antisepsis against *Candida* spp., including *C. parapsilosis*, and should be interpreted in the context of the surface carrier–based exposure model employed in this study, which more closely mimics antiseptic contact with abiotic hospital surfaces. Under these conditions, EtOH and CHG 0.5% produced the highest median reductions (≈4 log_10_, reaching up to 5 log_10_), confirming their strong fungicidal activity. The high efficacy of alcohol-based CHG formulations has been widely reported and is generally attributed to the rapid protein denaturation and membrane damage caused by alcohol, combined with the membrane-disrupting and residual activity of CHG, resulting in enhanced antimicrobial efficacy compared with aqueous CHG formulations [9,10,11]. Concentration-dependent killing by CHG against planktonic *Candida* cells and biofilms has been widely reported, with higher doses causing rapid membrane disruption and cytoplasmic leakage, leading to extensive cell death [35,36]. Likewise, EtOH has consistently demonstrated high activity against yeasts on hands and surfaces, supporting its role as a reliable agent for preventing healthcare-associated fungal transmission [11,43,44]. The better performance of EtOH compared with IPA observed in our assays agrees with previous reports showing that ethanol-based formulations exert more consistent fungicidal effects against *Candida* spp., whereas IPA may exhibit reduced activity when concentration or exposure time is insufficient to disrupt fungal membranes and metabolic integrity [35].

Intermediate efficacy was observed for CHG 1%, PHMGH, and PCMX. The biphasic antimicrobial behavior of CHG may explain the variability observed at this concentration, as formulation differences, exposure conditions and organic load can markedly influence its antifungal activity [45,46]. Aqueous CHG formulations are known to act more slowly than alcohol-based formulations, which provide rapid protein denaturation and immediate microbial killing; this difference may be especially relevant under surface carrier conditions with short exposure times, as used in the present study [10,11]. PHMGH demonstrated consistent antifungal activity against *Candida* spp., including on abiotic surfaces, corroborating previous reports describing this cationic polymer as an effective antimicrobial agent [26,30]. PCMX, commonly used in hand-wash formulations, also exhibits antifungal activity but is generally less potent than CHG and alcohols, particularly against non-albicans *Candida* species and established biofilms [35,47].

The lower efficacy of CHG 0.12% observed in this study is consistent with previous reports showing that sub-therapeutic CHG concentrations often result in only partial growth inhibition and fail to achieve high-level disinfection thresholds (≥4 log_10_ reduction), particularly in the presence of organic load or biofilm-like conditions [36,45]. PVP-I, H_2_O_2_, and TCS also exhibited limited activity (≤1.5 log_10_). The reduced efficacy of PVP-I in surface assays has been associated with decreased availability of free iodine in the presence of organic matter [35,48], while the variable activity of H_2_O_2_ against *Candida* spp. is partly attributed to catalase and oxidative stress response mechanisms, especially in biofilms [49]. TCS exhibits limited antifungal activity and is currently considered suboptimal for fungal control [35,50].

The efficacy hierarchy observed (EtOH/CHG 0.5% > PHMGH/PCMX/CHG 1% > CHG 0.12% > IPA/PVP-I/H_2_O_2_ > TCS) aligns with international infection-control literature, which emphasizes alcohol-based and adequately concentrated CHG formulations as the most reliable agents for preventing *Candida* transmission via hands, devices and environmental reservoirs [11,43,44]. Importantly, these findings demonstrate that antiseptic efficacy varies substantially depending on formulation and concentration, particularly under surface exposure conditions involving drying and organic matter. This reinforces that antiseptic performance observed in suspension tests does not necessarily translate to surface disinfection scenarios.

To complement these reductions and to guide the subsequent combination assays, MIC determinations were performed specifically for CHG and PHMGH. In these tests, CHG displayed a wide MIC range (2.4–620 ppm), reflecting the known inter-strain variability in *Candida* susceptibility. Such variation has been associated with differences in cell wall composition, induction of efflux pump systems, and enhanced cellular stress responses [35,36]. In contrast, PHMGH consistently exhibited the lowest MIC values (0.4–0.9 ppm for most clinical isolates; 1.9 ppm for *C. albicans* ATCC 10231 and 0.9 ppm for *C. parapsilosis* ATCC 90018), confirming its high antifungal potency against *Candida* spp. These results are consistent with previous reports showing PHMGH to exert strong membrane-disruptive activity at very low concentrations, leading to rapid loss of cell integrity and marked fungicidal effects in planktonic cells and on abiotic surfaces [26,37].

Among the phytochemicals, eugenol and thymol produced moderate MIC values (312–1250 ppm), outcomes consistent with multiple studies demonstrating that these phenolic terpenoids exert antifungal effects through membrane destabilization, ergosterol binding interference and induction of oxidative stress in *Candida* cells [51,52]. Eugenol has been shown to inhibit biofilm formation and to potentiate the activity of conventional antifungals such as fluconazole and amphotericin B, while thymol has demonstrated activity against non-*albicans Candida*, including *C. parapsilosis*, albeit at relatively high concentrations [51,53]. In contrast, eucalyptol (1,8-cineole) and menthol exhibited considerably weaker antifungal activity, with MICs reaching 80,000 ppm and 10,000–20,000 ppm, respectively. This limited efficacy agrees with the literature, which generally reports that monoterpene hydrocarbons and cyclic ethers, including cineole and menthol, display inferior antifungal potency compared with phenolic compounds, likely due to weaker interactions with fungal membranes and reduced ability to generate lethal oxidative stress [52,54].

Combination assays demonstrated that eugenol and menthol significantly enhanced the antifungal activity of CHG. Reductions in CHG MIC values ranged from 8-fold to over 300-fold depending on the isolate, with the highest reductions observed in a subset of clinical isolates rather than uniformly across all strains (see Table 1). This indicates that the magnitude of the synergistic effect was isolate-dependent. The observed MIC reductions are consistent with the known membrane-disrupting properties of phenolic and terpene compounds such as eugenol and menthol [51,52], which may increase fungal cell membrane permeability and facilitate the penetration of CHG, thereby enhancing its anti-fungal activity. This mechanistic explanation is supported by previous studies demonstrating that essential oil components can potentiate antimicrobial activity by altering membrane integrity.

This dose-sparing effect may also have important toxicological implications. In the *C. elegans* model, CHG toxicity was concentration-dependent, and the synergistic combinations substantially reduced the effective CHG concentrations required for antifungal activity. For combinations with eugenol and menthol, CHG concentrations in synergistic interactions ranged from 0.6 to 9.7 ppm, while for eucalyptol they ranged from 5 to 19 ppm, values below those associated with significant toxicity in the *C. elegans* model. In contrast, some thymol combinations resulted in CHG concentrations approaching 39 ppm, at which reduced survival was observed after prolonged exposure, indicating that toxicity may still be a concern depending on the final concentration used. These findings suggest that the primary advantage of the synergistic combinations may lie not only in enhanced antifungal activity but also in the potential reduction in antiseptic-associated toxicity.

By contrast, combinations involving thymol or eucalyptol displayed more heterogeneous outcomes. While synergism predominated for several isolates, indifferent interactions were frequently observed, particularly among reference strains, and consistent MIC reductions were not uniformly achieved. These variable responses may reflect compound-specific membrane interactions or adaptive stress responses that limit uptake or reduce susceptibility, phenomena previously described for certain essential oil constituents when applied at sublethal concentrations or in mixed formulations [52].

For PHMGH, interactions with phytochemicals were predominantly classified as indifferent across most *C. parapsilosis* clinical isolates and reference strains, with synergistic effects observed only in a limited number of strain-specific combinations. Notably, clear synergy was detected primarily for PHMGH–thymol associations in isolate 2A010 and in *C. parapsilosis* ATCC 90018, whereas other phytochemical combinations resulted mainly in unchanged MIC values and minimal fold reductions. This interaction profile is biologically plausible, as the extremely low MICs and strong membrane-disruptive activity of PHMGH may restrict the extent to which additional compounds can further enhance antifungal efficacy once near-maximal activity has already been achieved. Similar patterns have been reported for combinations involving highly potent biocides and natural products, where measurable synergy is uncommon and typically emerges only under specific strain- and concentration-dependent conditions [54].

In addition to antifungal efficacy, the toxicological profile of antiseptics represents a critical determinant for their safe and sustained use in healthcare settings. Using *C. elegans* as an in vivo model, our findings indicate that CHG exhibits a narrow margin between antifungal activity and biological tolerance, as concentrations above 39 ppm induced acute toxicity within the first hours of exposure. This observation is consistent with previous reports describing dose-dependent cytotoxic and irritant effects of CHG on eukaryotic cells, including epithelial and invertebrate models, particularly at concentrations exceeding those required for antimicrobial activity [55]. In contrast, PHMGH displayed a more gradual, concentration-dependent toxicological profile in *C. elegans*, with no evidence of acute early lethality across the tested concentration range [27,56].

This study has some limitations that should be considered when interpreting the results. The experiments were conducted under controlled laboratory conditions using a surface carrier model, and therefore clinical validation studies are still required to confirm the effectiveness of these antiseptics under real healthcare conditions. In addition, bio-film-specific assays were not included, which may influence antiseptic tolerance and should be addressed in future studies. Although a relatively large number of clinical isolates was included, all isolates were obtained from a specific clinical setting, which may limit the generalizability of the findings. Furthermore, toxicity was evaluated using the *C. elegans* model, which provides an initial in vivo assessment but does not directly represent human toxicity, and additional toxicological studies in mammalian models are necessary to better establish the safety profile of the tested compounds.

## 5. Conclusions

This study demonstrated that antiseptic efficacy against *C. parapsilosis* varies depending on the testing method and exposure conditions. Surface carrier assays identified 70% ethanol and 0.5% alcoholic chlorhexidine as the most effective agents under simulated surface contamination conditions, while PHMGH showed strong inhibitory activity but lower surface killing efficacy. The synergistic interactions observed between CHG and selected phytochemicals resulted in substantial reductions in effective MIC values, suggesting that combination strategies may enhance antifungal efficacy while reducing antiseptic concentration. These findings highlight the importance of using complementary methods to evaluate antiseptic performance and may contribute to improved infection control strategies in healthcare settings.

## Figures and Tables

**Table 1:** Fractional inhibitory concentration index (FICI) values for the combinations of chlorhexidine (CHG) with phytochemicals against 12 clinical isolates of *Candida parapsilosis* and two reference strains.

Isolate/Control (ATCC)	MIC (ppm)																
	ALONE					IN COMBINATION											
	CHG	EUC	EUG	MEN	THY	CHG EUC	FR CHG	FICI (Int.)	CHG EUG	FR CHG	FICI (Int.)	CHG MEN	FR CHG	FICI (Int.)	CHG THY	FR CHG	FICI (Int.)
A013	310	40,000	620	20,000	1250	9 10,000	34	1.61 (I)	9 39	34	0.09 (**S**)	0.6 312	64	0.02 (**S**)	4.8 2500	65	0.07 (**S**)
D011	310	40,000	1250	10,000	310	9 10,000	34	0.80 (I)	9 39	34	0.06 (**S**)	0.6 312	32	0.03 (**S**)	9.7 1250	32	0.13 (**S**)
A003	310	40,000	620	10,000	620	5 1250	62	0.20 (**S**)	5 78	62	0.14 (**S**)	155 312	32	0.05 (**S**)	39 620	8	0.14 (**S**)
D004	620	40,000	620	20,000	1250	9 1250	69	0.20 (**S**)	9 39	69	0.08 (**S)**	1.2 312	64	0.02 (**S**)	39 620	16	0.08 (**S**)
2A002	620	40,000	620	10,000	1250	2 5000	310	0.81 (I)	2 150	310	0.25 (**S**)	0.6 312	32	0.03 (**S**)	19 1250	33	0.06 (**S**)
2A010	310	40,000	1250	10,000	1250	2 20,000	155	1.60 (I)	2 150	155	0.13 (**S**)	0.6 312	32	0.03 (**S**)	19 1250	16	0.07 (**S**)
2D028	620	40,000	1250	20,000	1250	2 5000	310	0.40 (**S**)	2 150	310	0.12 (**S**)	0.6 312	64	0.02 (**S**)	19 1250	33	0.06 (**S**)
2D008	150	40,000	620	20,000	1250	1 5000	150	0.81 (I)	1 310	150	0.51 (**S**)	0.6 312	64	0.02 (**S**)	19 1250	8	0.13 (**S**)
3D021	310	40,000	2500	20,000	1250	2 1250	155	0.05 (**S**)	2 150	155	0.07 (**S**)	155 312	64	0.02 (**S**)	78 310	4	0.25 (**S**)
3A033	310	40,000	1250	10,000	2500	19 1250	16	0.10 (**S)**	19 19	16	0.08 (**S**)	1.2 312	32	0.03 (**S**)	78 310	4	0.25 (**S**)
3D033	6250	40,000	2500	10,000	2500	19 1250	329	0.05 (**S)**	19 19	329	0.01 (**S**)	1.2 312	32	0.03 (**S**)	39 620	160	0.07 (**S**)
3A086	310	40,000	2500	20,000	1250	5 10,000	62	0.40 (**S**)	5 78	62	0.05 (**S**)	0.6 312	64	0.02 (**S**)	19 1250	16	0.07 (**S**)
*C. albicans* ATCC 10231	620	80,000	1250	20,000	150	5 10,000	62	0.40 (**S**)	5 310	62	0.26 (**S**)	9.7 1250	16	0.06 (**S**)	4.8 150	129	0.87 (I)
*C. parapsilosis* ATCC 90018	310	40,000	625	20,000	78	5 10,000	62	0.40 (**S**)	5 150	62	0.26 (**S**)	9.7 1250	16	0.06 (**S**)	2.4 78	129	1.66 (I)

ppm: parts per million; CHG: Chlorhexidine digluconate (0.5% in alcoholic solution); EUC: eucalyptol; EUG: eugenol; MEN: menthol; THY: thymol; S = synergistic (FICI ≤ 0.5); I = indifferent (FICI > 0.5–4.0); FICI = Fractional Inhibitory Concentration Index; Int., interaction type (synergistic, indifferent, or antagonistic); FR (fold reduction).

**Table 2:** Fractional inhibitory concentration index (FICI) values for the combinations of polyhexamethylene guanidine hydrochloride (PHMGH) with phytochemicals against 12 clinical isolates of *Candida parapsilosis* and two reference strains.

Isolate/ Control (ATCC)	MIC (ppm)																
	ALONE					IN COMBINATION											
	PHMGH	EUC	EUG	MEN	THY	PHMGH EUC	FR PHMGH	FICI (Int.)	PHMGH EUG	FR PHMGH	FICI (Int.)	PHMGH MEN	FR PHMGH	FICI (Int.)	PHMGH THY	FR PHMGH	FICI (Int.)
A013	0.9	80,000	625	20,000	312	0.9 80,000	1	2.0 (I)	0.9 625	1	2.0 (I)	0.9 20,000	1	1.0 (I)	0.9 78.12	1	1.25 (I)
D011	0.9	80,000	625	10,000	312	0.9 80,000	1	2.0 (I)	0.9 625	1	2.0 (I)	0.9 10,000	1	1.0 (I)	0.9 78.12	1	1.25 (I)
A003	0.9	80,000	625	10,000	312	0.9 40,000	1	1.5 (I)	0.9 625	1	2.0 (I)	0.9 10,000	1	1.0 (I)	0.9 78.12	1	1.25 (I)
D004	0.9	80,000	625	20,000	312	0.9 80,000	1	2.0 (I)	0.9 625	1	2.0 (I)	0.9 20,000	1	1.0 (I)	0.9 78.12	1	1.25 (I)
2A002	0.4	80,000	625	10,000	312	0.9 40,000	0.4	2.7 (I)	0.9 625	0.4	3.2 (I)	0.9 10,000	0.4	1.0 (I)	0.9 156.2	0.4	2.75 (I)
2A010	0.9	80,000	312	10,000	625	0.9 40,000	1	1.5 (I)	0.9 312	1	2.0 (I)	0.9 1250	1	0.12 (S)	0.002 39.06	500	0.06 (**S**)
2D028	0.9	40,000	625	20,000	312	0.9 40,000	1	2.0 (I)	0.9 625	1	2.0 (I)	0.9 20,000	1	1.0 (I)	0.9 78.12	1	1.25 (I)
2D008	0.9	80,000	625	20,000	625	0.9 80,000	1	2.0 (I)	0.9 625	1	2.0 (I)	0.9 20,000	1	1.0 (I)	0.9 156.2	1	1.25 (I)
3D021	0.4	80,000	625	20,000	312	0.9 80,000	0.4	3.2 (I)	0.9 625	0.4	3.2 (I)	0.9 20,000	0.4	1.0 (I)	0.9 156.2	0.4	2.75 (I)
3A033	0.9	80,000	625	10,000	625	0.9 80,000	1	2.0 (I)	0.9 625	1	2.0 (I)	0.9 10,000	1	1.0 (I)	0.9 78.12	1	1.12 (I)
3D033	0.9	40,000	625	10,000	625	0.9 40,000	1	2.0 (I)	0.9 625	1	2.0 (I)	0.9 5000	1	0.5 (**S**)	0.9 312.4	1	1.50 (I)
3A086	0.4	80,000	625	20,000	625	0.9 40,000	0.4	2.7 (I)	0.9 625	0.4	3.2 (I)	0.9 20,000	0.4	1.0 (I)	0.9 78.12	0.4	2.37 (I)
*C. albicans* ATCC 10231	1.9	80,000	1250	20,000	156	0.9 4000	2.1	1.0 (I)	0.9 625	2.1	1.0 (I)	0.9 20,000	2.1	1.0 (I)	0.9 156.2	2.1	1.47 (I)
*C. parapsilosis* ATCC 90018	0.9	40,000	625	20,000	1250	0.9 10,000	1	1.2 (I)	0.9 312	1	1.5 (I)	0.9 20,000	1	1.0 (I)	0.002 39.06	500	0.03 (**S**)

ppm: parts per million; EUC: eucalyptol; EUG: eugenol; MEN: menthol; THY: thymol; S = synergistic (FICI ≤ 0.5); I = indifferent (FICI > 0.5–4.0); FICI = Fractional Inhibitory Concentration Index; Int., interaction type (synergistic, indifferent, or antagonistic); FR (fold reduction).

## Data Availability

The original contributions presented in this study are included in this article. Further inquiries can be directed to the corresponding authors.

## Abbreviations

The following abbreviations are used in this manuscript:

MIC	Minimum inhibitory concentration
CHG	Alcoholic chlorhexidine 0.5%
PHMGH	Polyhexamethylene guanidine hydrochloride
EtOH	Ethanol 70%
H_2_O_2_	Hydrogen peroxide 3%
IPA	Isopropyl alcohol 70%
PCMX	Chloroxylenol
PVP-I	Povidone–iodine
TCS	Triclosan 0.5%
RAPD	Random Amplified Polymorphic DNA
SADH	Secondary alcohol dehydrogenase
MALDI	Matrix-assisted laser desorption/ionization
TOF	Time-of-flight
ATCC	American type culture collection
YPD	Yeast peptone dextrose
CFU	Colony-forming units
RF	Logarithmic reduction factor
FICI	Fractional inhibitory concentration index
NGM	Nematode growth medium
DMSO	Dimethylsulfoxide
ANOVA	Analysis of variance
ppm	Parts per million
